# Safety and efficacy of varicella vaccination in children with JIA treated with biologic therapy

**DOI:** 10.1186/1546-0096-12-S1-O20

**Published:** 2014-09-17

**Authors:** Natasa Toplak, Tadej Avcin

**Affiliations:** 1 Department of Allergology, Rheumatology and Clinical Immunology, University Children's Hospital Ljubljana, University medical centre Ljubljana, Ljubljana, Slovenia

## Introduction

Varicella infection is a highly contagious disease which can have a complicated course especially in immunocompromised children and children receiving immunomodulatory therapy.

## Objectives

to evaluate safety and efficacy of varicella vaccination in children with JIA treated also with biologic therapy.

## Methods

We designed a prospective study on a long term follow up. Five patients with JIA (median age 5, range 3,5-7 years), treated also with biologic therapy (2 etanercept, 2 tocilizumab, 1 infliksimab), received 2 doses of varicella vaccine. One patient treated with etanercept received the first dose before starting etanercept, others received both doses on biologic therapy. Before vaccination JIA was stable on therapy and lymphocytic populations (CD3, CD4, CD8, CD19, NK) were normal in all vaccinated children. All had negative varicella serology. Parents were asked for written informed consent before vaccination. After vaccination children were followed for disease activity, infections and protective antibodies (pAb) against varicella virus on a long term.

## Results

There were no serious side effects after vaccination and no clinical varicella infection in a period of 3 months after vaccination. One patient had mild local reaction after the first dose. One patient treated with etanercept got adenovirus infection 3 days after the second dose of vaccine. Disease activity remained stable in all patients in a period of three months after the second dose. Four patients (75%) had pAb against varicella virus 6 weeks after the second dose. One patient treated with infliximab and methotrexate didn’t develop pAb after the second dose. Two patients treated with etanercept developed low levels of pAb after the second dose. Two patients treated with tocilizumab developed low levels of pAb even after the first dose and very high levels of pAb after the second dose. One patient treated with etanercept got varicella infection 4 months after the second dose despite the protective, however, low levels of pAb. Varicella infection was mild. The other patient treated with etanercept lost his pAb 22 months after the second dose. One patient treated with tocilizumab still has low pAb 27 months after the second dose and one patient treated with tocilizumab has very high pAb 3 month after the second dose.

## Conclusion

Varicella vaccination appears to be safe in JIA patients treated also with biologic therapy. However, it is possible that protection is only short term and does not always protect against infection. Follow up of pAb is recommended and the need for revaccination should probably be carefully considered in cases with high risk of varicella infection. Larger cohort studies are needed to obtain more reliable data on varicella vaccination in JIA patient treated with biologic therapy.

## Disclosure of interest

None declared.

